# Teachers’ reported practices for teaching writing in England

**DOI:** 10.1007/s11145-015-9605-9

**Published:** 2015-11-21

**Authors:** Julie E. Dockrell, Chloë R. Marshall, Dominic Wyse

**Affiliations:** Department of Psychology and Human Development, UCL Institute of Education, University College London, 20 Bedford Way, London, WC1H 0AL UK

**Keywords:** Teaching writing, England, Primary school, Elementary school, Simple view of writing

## Abstract

To date there have been no systematic studies examining the ways in which teachers in England focus and adapt their teaching of writing. The current study addresses this gap by investigating the nature and frequency of teachers’ approaches to the teaching of writing in a sample of English primary schools, using the ‘simple view of writing’ as a framework to examine the extent to which different aspects of the writing process are addressed. One hundred and eighty-eight staff from ten different schools responded to an online questionnaire. Only the data from class teachers (n = 88) who responded to all items on the questionnaire were included in the final analyses. Respondents enjoyed teaching writing and felt prepared to teach it. However, despite feeling that they were effective in identifying approaches to support students’ writing, nearly half reported that supporting struggling writers was problematic for them. Overall teachers reported more work at word level, occurring several times a week, than with transcription, sentence or text levels, which were reported to occur weekly. Planning, reviewing and revising occurred least often, only monthly. For these variables no differences were found between teachers of younger (age 4–7) and older students (age 8–11). By contrast, an examination of specific aspects of each component revealed differences between the teachers of the two age groups. Teachers of younger students focused more frequently on phonic activities related to spelling, whereas teachers of older students focussed more on word roots, punctuation, word classes and the grammatical function of words, sentence-level work, and paragraph construction.

## Introduction

Writing is a higher order skill that develops over time through interactions between the child’s skills and cognitive resources, the instructional context, and the demands of the writing task (Kellogg, 2008). Significant advances have been made in our understanding of the developing components of text production (Wagner et al., 2011), the demands placed on the cognitive system to produce written text (Dockrell & Connelly, 2015), and which writing interventions are effective (Graham, Gillespie, & McKeown, 2013). There is a close link between classroom teaching and the writing produced by students (Fisher, Myhill, & Twist, 2011), and instructional quality has been shown to be uniquely related to children’s written composition over and above child-level predictors (Kim, Al Otaiba, Sidler, & Gruelich, 2013). However, there is limited information about the ways in which mainstream teachers approach the teaching of writing. To date there have been no systematic studies examining the ways in which teachers in England focus and adapt their teaching of writing across the primary school phase of education. The current study aims to address this gap by examining the nature and frequency of teachers’ approaches to the teaching of writing in a sample of English primary schools.

### Teachers teaching writing

A number of studies, primarily from the USA, have described teachers’ practices with respect to teaching writing (Cutler & Graham, 2008; Graham, Harris, Fink-Chorzempa, & MacArthur, 2003; Richards, Sturm, & Cali, 2012), and for specific components of writing such as spelling (Graham et al., 2008b) and handwriting (Barnett, Stainthorp, Henderson, & Scheib, 2006; Graham et al., 2008a). Some of these studies have also considered the impact of instruction for struggling writers (Graham et al., 2003; Richards et al., 2012). Studies have varied as to whether they targeted one specific year group (Kim et al., 2013) or several year groups (Richards et al., 2012), the number of teachers who participated (N = 10–220 completing questionnaires or interviews), and whether respondents were targeted strategically or were representative of a random sample of school teachers (see for example Graham, Capizzi, Harris, Hebert, & Morphy, 2014). Methods to elicit teachers’ views have also differed. In some cases teachers have been interviewed, others have completed surveys; moreover, surveys have varied in the questions asked and the types of responses required.

Despite these differences in samples and survey questions, a number of general findings are evident. Writing instruction varies considerably across school settings and in the amount of time that teachers allocate to writing instruction (Cutler & Graham, 2008; Graham, Harris, & Chorzempa, 2002; Richards et al., 2012). For example, in one of the largest samples of teacher respondents, Applebee and Langer (2011) found that students in middle and high schools were not engaged in much extended writing and only 50 % of the observed English classes included specific writing-related instruction. The authors concluded that in these ‘best case scenarios’ students would have on average just over 3 min of instruction a day related to explicit writing strategies. Similarly, Richards et al. (2012) reported on the nature and frequency of 107 first, third and fifth grade general education teachers in Michigan and found significant variation in the teachers’ reported practices. Despite this variation there was indicative evidence of changes across grade for both activities and instructional practices.

More fine-grained analyses have pointed to subtle differences in the nature and frequency of writing-related activities. Elementary school teachers focused, on average, several times a week on basic skills, whereas planning, reviewing and revising happened less frequently and the use of information technology for writing was rare (Graham et al., 2003). In middle school (students aged 11–13) teachers reported using a variety of evidence-based practices but they applied most of those practices infrequently (Graham et al., 2014). Moreover, teachers often found teaching writing challenging and reported being inadequately prepared to teach writing (Graham et al., 2008a, 2008b, 2014).

The extent to which these conclusions can be generalized internationally is currently unclear. Teacher training differs across countries, the use of national curricula varies, and the emphases placed on the key purposes and processes in learning to write differ, and are often subject to local guidelines. Furthermore, teachers are reported to change instruction practices based on the standards that are set (Gross, Kirst, Holland, & Luschei, 2005). Under current law in England, children must be in education from the term of their fifth birthday. National curricula in England include the Early Years Foundation Stage (EYFS, for children aged three to five) and the national curriculum (for children aged five to 11). The curriculum content has varied as a result of political change, but English, and writing as a component of English, continue to be part of the current national curriculum (Department for Education, 2013).

The national curriculum is organised into ‘key stages’, which for primary school children comprises Reception/Key Stage 1 (R/KS1; ages 4–7) and Key Stage 2 (KS2; ages 7–11). At the time of the study the national curriculum for English in primary schools included speaking, listening, reading and writing (DfEE & QCA, 1999). During KS1 students are taught to communicate meaning in both narrative and non-fiction texts and to spell and punctuate correctly. In addition to the curricular elements in KS1, during KS2 students learn the main rules and conventions of written English including the grammar of complex sentences. They also start to explore how the English language can be used in different ways to express meaning. At this point they also are expected to use planning, drafting and editing to improve their fiction and non-fiction texts.

When children are in Year one (aged five to six) their reading is assessed through a national phonics screening check. Until 2015, teacher assessments occurred in English at the end of KS1 and national tests in English (including reading and writing) occurred at the end of KS2. Assessment of writing in primary classrooms is based on teachers’ ongoing assessments. At the end of KS1 statutory national curriculum tasks and tests must be used to inform final teacher assessment judgments, including for writing. At the end of KS2, schools report externally-moderated teacher assessment of pupil outcomes to the Standards and Testing Agency, including for writing. There are also externally marked national curriculum tests including a test of English grammar, punctuation, and spelling at the end of KS2. The programme of study for writing is divided between transcription (spelling and handwriting) and composition (Department for Education, 2013). Two statutory appendices on spelling and on vocabulary, punctuation, and grammar provide an overview of the specific features that should be included in the teaching programmes. Thus, in England there is clear and explicit guidance about what should be taught and what is assessed.

In England, children perform less well in writing compared to other subjects (DfE, 2012), and this has been a topic of continued concern. Two studies carried out in England, very different in kind, examined teachers’ reported practices in teaching writing. Fisher and Twist (2011) carried out a small number of interviews with teachers of Year 3 children (age eight) and Year 4 children (age nine). Their focus was on the success, or otherwise, of the government’s ‘Every Child a Writer’ programme, and these are the only data on the teaching of writing in England reported by the government (Department for Education, 2012). As such the data cannot be generalized to schools not involved in the Every Child a Writer programme, and importantly the interviews were only used to support the report’s general conclusions that the programme showed little effect. Whole class lessons, variation in adherence to lesson plans and weak subject knowledge characterized the results obtained. By contrast, Barnett et al. (2006) focused only on handwriting and collected data via a teacher questionnaire (N = 39). The results demonstrated that although the majority of teachers considered handwriting to be an important skill, there was significant variation in school policies for the teaching of handwriting, many teachers were not well prepared to teach it, and there was little time for children to practise.

The complexity of the writing process places significant demands on teachers’ expertise and teaching time. There are a range of key skills that need to be taught and a range of different ways in which teaching can occur. To help teachers structure what is taught and how it should be taught a framework outlining the writing process could inform practice. A developmental model of the writing processes provides an understanding of writing development and has the potential to identify developmental differences and points for instruction.

### What to teach

**Fig. 1:**
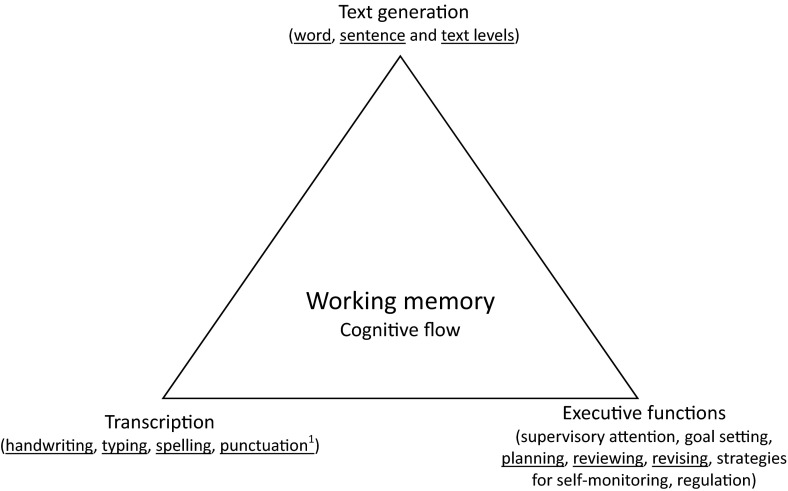
The simple view of writing (adapted from Berninger et al., 2009). The components of the model investigated in the current study are *underlined*. ^1^Although punctuation is not included in the original or revised model, we include it within transcription as it has to do with mark-making (see also Hayes & Olinghouse, 2015)

A prerequisite to teaching children to write is an understanding of the skills that are developing as children learn to write, as these can be the focus of instruction. The multiple components of the developing writing process have been captured in the ‘simple view of writing’ and the more recent ‘not-so-simple view of writing’ (see Berninger, Garcia, & Abbott, 2009 for a review). The model synthesizes diverse traditions in compositional research whereby writing development can be represented, figuratively, as a triangle in a working memory environment in which transcription skills (handwriting/typing and spelling) and executive functions (e.g. planning, reviewing and revising) are the vertices at the base that enables the goal of text generation (the top of the triangle) to proceed efficiently (see Fig. 1, based on Berninger et al., 2009). From an instructional perspective the model captures three different components of writing: transcription, text generation and executive functions. Within each of these components there are separate domains which can be the focus of instruction. For example text generation includes word, sentence and text level work and transcription includes spelling and handwriting/word processing. Within each domain separate skills could be the focus of instruction; for example, text level work could include instruction in paragraph construction or focussing on topics and ideas.

In the model, writing development is described as the product of the development of transcription (spelling and handwriting), text generation skills at word, sentence and text level, and executive functions including planning, reviewing and revising (Berninger, 2000; Berninger et al., 2002). The applicability of the ‘simple view of writing’ framework to educational settings is evident as these different domains of the writing process can be conceptualised as reflecting different potential foci for teaching writing, where the emphasis should reflect the child’s writing skills. Thus in the initial stages of learning to write and for some struggling writers one would expect a greater focus on lower level writing skills such as the transcription skills of spelling and handwriting and word-level work, whereas when the child becomes a more proficient producer of texts emphasis might be placed on higher level writing skills such as planning and the more complex aspects of sentence grammar. A focus on these domains of written text production at different levels of writing proficiency is also supported by research evidence. There is strong evidence from experimental and quasi-experimental studies that directly teaching spelling, vocabulary and sentence-level skills improves writing performance (Institute of Education Sciences, 2012). A key recommendation is to provide children with daily opportunities to write (Graham & Perin, 2007c).

The vertices of the simple view of writing model, thus, provide a framework to examine the extent to which teachers focus on these different components of the writing process. Which aspects should be included within the respective domains requires further elaboration. For example, word-level work which focusses on phonics is probably best considered in relation to spelling and therefore the transcription component (Galuschka, Ise, Krick, & Schulte-Korne, 2014). By contrast, word-level work which focuses on word meaning and semantic associations may underpin text generation (Dockrell & Connelly, 2015).

### How to teach

There are a number of programmes available in the UK to support the teaching of writing, although few have been subject to rigorous evaluation (Department for Education, 2012). By contrast, meta-analyses point to the effectiveness of specific pedagogical practices that do enhance writing skills (Andrews, Torgerson, Low, & McGuinn, 2009; Graham & Perin, 2007a; Hebert, Gillespie, & Graham, 2013; Tracy, Reid, & Graham, 2009). Encouraging students to write for audiences and purposes and regular and extensive shared, guided and independent writing have been shown to be effective (Graham et al., 2013; Graham, Harris, & Hebert, 2011; Institute of Education Sciences, 2012). In the review by Andrews et al. (2009), explicit scaffolding of writing processes improved text production, self-motivation and reasoning. As Andrews et al. (2009) show, supporting children’s writing in this way can be done using a number of different devices, but explicit contrasts and feedback are crucial. Both collaborative writing, where writers work together to plan, draft, revise and edit their compositions (Graham & Perin, 2007b), and paired writing (Yarrow & Topping, 2001) are effective in supporting students’ writing. The extent to which these are currently embedded in classroom practice in England is not clear.

### Current study

To the best of our knowledge, and despite the concerns that have been reported in England and internationally about the development of children’s writing skills, no recent study has systematically asked teachers about their teaching of writing in England (see Cato, Fernandes, Gorman, Kispal, & White, 1992 for some early descriptive work). Our research questions were the following:

1. How do primary school teachers in England feel about their preparation for teaching writing, and what training have they received?
2. Which components of the Simple View of Writing do teachers focus on
  - How frequently does this occur?
  - Is this frequency related to children’s educational phase (key stages)?
  - Are there significant differences in the amount of time reported to be devoted to different components within the Simple View of Writing?
3. What teaching practices are used to support the development of children’s written text, does this differ between educational phases and do teachers use any specialised programmes to support their teaching?

Teachers were invited to complete an on-line questionnaire. The first research question was addressed by describing the responses. To answer the third research question, teachers’ reports of different teaching practices between the two key stages were compared. To explore the second research question we reasoned that the curriculum should cover each component of the model underlined in Fig. 1 and the domains captured within these. For each component we identified the domains specified within the model and items related to each domain from both the research literature and the national curriculum. We predicted that there would be differences in emphasis between the two key stages, with a greater emphasis on transcription and word-level work (lower level writing skills) at KS1 and a greater emphasis on planning, reviewing and revising and text-level work (higher level writing skills) at KS2.

## Methods

### Participants

#### Schools

Ten mainstream primary schools from urban areas in London and the South East of England that were part of a larger evaluation project (see Dockrell, Marshall, & Wyse, 2015) took part in the survey. Completion of the questionnaire was encouraged by the head teachers in school but was voluntary. Schools did not provide data on the numbers of teachers and specialist support available at the time of the project. Eight of the schools were community schools, one voluntary aided and one an academy. Ofsted^1^ reports, which had been carried out in the last 3 years, indicated that eight of the schools received a good rating and two required improvement. Four schools included students aged between four and 11, one school for students aged between 3 and 11, three schools for students aged between 7 and 11, and two schools for students aged between four and seven. The mean school size was 360 students (*SD* = 129, range 184–625).

The data were collected prior to the evaluation project starting. None of the schools reported following any special writing programmes; all followed the National Curriculum in place at the time (the 2012–2013 school year). Numbers of respondents varied by school (range 2–40), partly reflecting the different sizes of the schools.

#### Students

Student data from nationally-collected metrics were used to profile the schools. At the time of data collection (January 2013) school census data was collected annually by the government, recording students who were eligible to receive free school meals (an index of deprivation^2^), students for whom English was an additional language, and students with special educational needs (SEN). Compared to national data available for the year of data collection, there were more students receiving free school meals (project schools 23 %; national average 17 %), more students with SEN with a statement or on school action plus (project schools 20 %; national average 9 %), but fewer students recorded with English as an additional language (project schools 12 %; national average 18 %). In addition the teachers were teaching students who were performing below national averages. Fewer students met national targets in the national assessments completed at the end of primary school that were in use at the time: writing (project schools 53 %; national average 78 %), reading (project schools 68 %; national average 86 %) and speaking and listening (project schools 60 %; national average 83 %).

#### Respondents

The questionnaire was completed by 188 staff across the schools, of whom 14 had only management roles and a further 12 had specialist cross-school roles. The remaining 162 respondents had teaching roles with the students. Sixty-six respondents were teaching assistants or worked in specialist resources. The remaining 106 were class teachers; 45 were based in reception and KS1 classrooms and 61 in KS 2 classrooms, and they came from all the schools in the project. Analyses used data from the classroom teachers as the bases for comparisons. The majority of the teachers were female (87 %), which is commensurate with national data (female primary school teachers 87 %, DfE, 2010). Eighty-nine per cent of teachers had taught for more than 1 year and 19 % had taught for more than 15 years.

All respondents had a teaching qualification (Bachelor in Education 41 %, Post Graduate Certificate in Education 58 %, Masters in Education 1 %). Sixty-four per cent of the respondents reported having an honours degree, reflecting similar levels of degree and higher qualifications to the national pattern for primary school teachers in England (59 %, DfE, 2010).

### Questionnaire

A questionnaire was designed following a review of effective practices reported in the research literature, a review of current government recommendations for the teaching of writing, a review of previous studies with school staff, and three focus groups with experienced practitioners. The focus groups provided information to develop the rating scale that they felt reflected practice in schools in England that were following the national curriculum. The questionnaire was piloted with a group of literacy support teachers (N = 28), and amendments were made to clarify questions using language of the national curriculum e.g. ‘morphology’, ‘phonemes’, ‘prefixes’ and ‘suffixes’ (see English programmes of study KS1 and 2, Department for Education, 2013), to restrict the rating scale to six points and to extend open-ended options as required for each section in the questionnaire.

The final questionnaire consisted of five sections. The first section asked teachers to identify their school, provide demographic information including gender, age bracket (in 10 year gaps), qualifications, their role in the school and the year group(s) they were currently teaching. Classroom teachers were also asked to report on the numbers of students in their class and the curriculum levels they were working at.

The second section included one Likert scale question examining preparation to teach writing and eight statements related to the teaching of writing where respondents rated their response on a six point Likert scale from ‘strongly agree’ to ‘strongly disagree’ e.g. ‘I am effective at teaching writing’. This section finished with a question about attendance at professional development activities to teach writing. Where respondents had attended such activities they were asked indicate which ones from a checklist. It was also possible for respondents to add to the list of activities in response to a final open-ended question.

The third section contained the items related to the components of the Simple View of Writing and teaching practices. Prior to the third section respondents were provided with the following instructions:

‘You will be presented with a list of activities that some teachers use to support written text production. Five areas of writing are covered: handwriting, spelling, punctuation, composition, and planning, reviewing and revising. The use and frequency of use of these approaches will depend on the age group you teach and the students in your class. It is not expected that you will use all these activities. Please indicate whether you use these activities for your current class and if you use them, the frequency of their use. The order of the questions DOES NOT imply a sequence of teaching,’

Respondents were then asked three questions about handwriting and word processing, nine questions about spelling, four questions about punctuation, 14 questions related to composition of text at word, sentence and text level including four questions related to grammar, and seven questions related to planning, reviewing and revising. To ensure that no potential issues had been missed, at the end of each set of questions there was an opportunity for respondents to specify any alternative approaches that they used or to further clarify their responses.

The fourth section included 13 teaching practices that might be used to support the development of children’s writing. These practices were drawn from the research literature and the national curriculum, but additionally from writing programmes that were available in the UK at the time. Respondents were also asked to list in an open-ended question any published schemes that they used to support children’s writing. A final section, which is not reported here, focussed on assessments to evaluate students’ progress in writing (but see Dockrell et al., 2015).

Items about the content of teaching were rated on a five-point Likert scale with 5 indicating that a topic was taught daily, 4 several times a week, 3 once a week, 2 monthly, 1 several times a year and 0 not taught. The questionnaire is available from the corresponding author.

### Procedure

Ethical approval following the British Psychological Society guidelines was secured for an anonymised online questionnaire. The questionnaire was created using SurveyMonkey^®^ (https://www.surveymonkey.com/). Head teachers of the schools were approached by e-mail and phone and agreed to participate in the project. The head teacher decided the staff member responsible for disseminating the link to the questionnaire to the staff that they felt were appropriate to complete the questionnaire. Given the choices were made by the head teacher it is not possible to report completion rate per school. Schools provided staff with time to complete the questionnaire. Completion of the questionnaire was voluntary.

### Data cleaning and management

All respondents completed sections related to their training, general approach to the teaching of writing, students in their classrooms, and writing assessment. A small number of respondents left items about specific teaching practices incomplete but often provided a narrative response such as ‘I only teach when I feel children are ready’ or ‘I do these when children enter appropriate phases’. These respondents were removed from the analysis of what was taught (*n* = 18; Reception/KS1 *n* = 8 and KS2 *n* = 10). There were no significant differences between respondents who completed the full questionnaire and those who had missing items for gender [*χ*^2^(1, *N* = 106) = 1.54, *ns*], age category [*χ*^2^(4, *N* = 106) = 6.66, *ns*], key stage in which they were teaching [*χ*^2^(1, *N* = 106) = .85, *ns*], number of years of teaching category [*χ*^2^(3, *N* = 106) = .75, *ns*] or whether they had attended professional development activities related to writing [*χ*^2^(1, *N* = 106) = .95, *ns*]. However there were significant differences in the number of completed questionnaires by school [*χ*^2^(9, *N* = 106) = 23.52, *p* = .005]. Examination of the raw data indicated that for eight of the 10 schools questionnaires were fully completed by between 70 and 100 % of the respondents. However for two schools there were fully completed questionnaires for only 50 % of the respondents, one was an infant school where there were only two respondents and the other an infants and junior school. The schools did not differ in any other obvious way from schools where there were higher numbers of fully completed questionnaires.

Respondents had an option to record ‘not applicable to my teaching’ and these items were given a score of 0, i.e., not taught. The final sample included 88 teachers who completed all the items in the questionnaire.

## Results

The results are presented in three sections, reflecting our research questions. “Teachers’ preparation for and views about teaching writing” section provides data on the teachers’ views about teaching writing and the training they have received. “Domains of teaching, in relation to the components of the simple view of writing” section provides the teachers’ views on the components of the writing process targeted and the frequency with which this is done. “Reported teaching practices to support writing development” section reports the teachers’ approaches to the teaching of writing and their use of specialised programmes.

### Approach to analyses

Data from items with a nominal response format, school demographics, and responses to open-ended questions are presented descriptively. Results to questions using Likert scales were analysed using parametric statistics with the following provisos: all items were tested for skewness and kurtosis (Glass, Peckham, & Sanders, 1972). For each item analysed, we present the percentage of respondents who stated that they never did this activity. Where items met the conditions for normality and equal variance they were included in the parametric analyses and a stringent alpha level of .01 was used to ensure the reliability of the findings (Glass et al., 1972). Cronbach’s alpha coefficient for internal consistency reliability is presented for all domain subscales, and in all cases reliability coefficients are at least acceptable (>.7).

Exploratory analyses using MANOVA for each domain and, where appropriate, post-test comparisons were first computed to provide a preliminary analysis of responses. To allow robust comparisons across domains a mean value for each domain was computed which resulted in a composite scale value for that domain (Carifio & Perla, 2007).

### Teachers’ preparation for and views about teaching writing

Fifty-nine per cent of the respondents reported their training to teach writing as either very good or outstanding, with the remaining reporting that it was adequate. Ninety per cent of respondents reported either agreeing or strongly agreeing that they liked to teach writing and as a group (90 %) agreed they were effective teachers of writing. Indeed, only one respondent disagreed with this statement. Twenty-nine per cent agreed with the statement that teaching writing was challenging but overall (79 %) felt they were effective in identifying approaches to support students’ writing. However, 45 % reported that supporting struggling writers was a problem for them, and 59 % reported that there were limited resources to support children’s writing.

The majority (81 %) of respondents had attended professional activities related to writing. Respondents were able to record more than one form of training. The forms of training identified were typically in-service training (60 %), staff writing workshops (52 %), and specific courses on the teaching of writing (49 %). There were only three respondents who provided further information in the open-ended section: reading recovery, using speech and language therapists to support sentence structure work, and one specific training programme.

We examined whether teachers’ experience of training influenced their views on the challenges posed by lack of resources and teaching struggling writers. There were no significant differences between teachers who had attended training and those who had not in terms of their views of resources, χ^2^(4) = 8.06, *p* = .09. However those who had attended training courses were less likely to report that supporting struggling writers was a problem for them, χ^2^ (4) = 10.12, *p* = .04.

### Domains of teaching, in relation to the components of the simple view of writing

#### Transcription

*Handwriting and typing* We initially examined the frequency with which teachers reported supporting handwriting or typing. Practice in handwriting (cursive or printing) was reported to occur on average weekly across both reception/KS1 and KS2, although two per cent of respondents reported that they never did this (M R/KS1 = 3.00, SD 1.78; M KS2 = 3.12, SD = 1.51) while supporting children in typing was a much rarer occurrence that happened on average only annually and 15 per cent of respondents reported that they never did this (M R/KS1 = 1.16, SD .69; M KS2 = 1.14, SD = 1.04).

Only four respondents provided further information in the open-ended section: one indicating that some children get training in touch typing outside of school, two stating touch typing was done when ‘they are ready’ and a final respondent stating that touch typing was done once at the beginning of the academic year.

*Spelling* There were eight items that examined teachers’ focus on spelling, and Cronbach’s alpha was .74 for these items. Table 1 provides means (SDs) by Key Stage for reported spelling foci. As the table shows, sounding out phonemes occurred on average several times a week whereas explicit instruction of word families occurred monthly. An initial MANOVA examined year group differences across the spelling items. There was a statistically significant difference in teachers’ reported focus on the items by year groups taught, *F*(8, 79) = 10.94, *p* < .001; Wilk’s Λ = 0.47, partial eta squared = .53. Subsequent univariate testing indicated that teachers in reception/KS1 reported more frequently focussing on sounding out phonemes, which was reported to be happening virtually daily, *F*(1, 86) = 42.66, *p* < .001, partial eta squared = .33. By contrast, teachers in KS2 reported a greater focus on explicit instruction of morphology, and teaching was reported to occur weekly (explicit instruction of word families, roots and origins, *F*(1, 86) = 10.74, *p* = .002, partial eta squared = .11; explicit instruction in the use of suffixes and prefixes, *F*(1, 86) = 31.74, *p* < .001, partial eta squared = .27; explore the meaning, use and spelling of affixes, *F*(1, 86) = 10.52, *p* = .002, partial eta squared = .11). For the other four items related to spelling (analyse words into subcomponents, apply knowledge of spelling conventions, analyse knowledge of orthographic patterns and explicit instruction in the use of terminology) there were no significant differences between the key stages (all *p*s > .05). A mean score for the eight spelling items was computed, reflecting the frequency with which teachers reported focussing on spelling in their teaching of writing. All the teachers reported focussing on spelling and, on average, this was occurring weekly, but there was large variation across the respondents (M = 3.00, SD = .84).

**Table 1:** Mean (SDs) of teachers’ reported frequency for teaching spelling (0 = not taught to 5 taught daily)

	Reception and KS1 (*n* = 37)	Key stage 2 (*n* = 51)	Total (N = 88)	Percentage reporting not taught
Sound out phonemes*	4.92 (0.28)	3.53 (1.27)	4.11 (1.20)	0
Analyse words into subcomponents	2.57 (1.37)	3.08 (1.18)	2.86 (1.28)	1.1
Apply knowledge of spelling conventions	3.38 (1.53)	3.67 (1.14)	3.55 (1.32)	2.3
Analyse knowledge of orthographic patterns	3.65 (1.65)	3.27 (1.04)	3.43 (1.34)	2.3
Explicit instruction of word families, roots and origins*	1.86 (1.32)	2.76 (1.24)	2.39 (1.34)	2.3
Explicit instruction in the use appropriate terminology	2.59 (1.57)	3.25 (1.45)	2.98 (1.53)	3.4
Explicit instruction in the use of suffixes and prefixes*	1.49 (1.04)	2.78 (1.08)	2.24 (1.24)	4.5
Explore the meaning, use and spelling of affixes*	1.97 (1.28)	2.80 (1.11)	2.45 (1.25)	1.1

* Significant differences in teachers’ reported frequency of teaching for the two groups of students, *p* < 0.01

Seven respondents provided further information in the open-ended section. Of these, four mentioned doing activities when pupils were ‘ready’ and three specified programmes that they used to support spelling.

*Punctuation* There were four items that examined teachers’ targets for punctuation, and Cronbach’s alpha was .83. Table 2 provides means (SDs) by key stage for reported punctuation targets. As the table shows, teachers reported focussing on punctuation at the end of sentences weekly but focussing on speech marks only monthly. An initial MANOVA examined year group differences across the punctuation items. There was a statistically significant difference in teachers’ reported focus by year groups taught, *F*(4, 83) = 9.12, *p* < .001; Wilk’s Λ = 0.70, partial eta squared = .31. Subsequent univariate testing indicated that teachers in KS2 reported a greater focus on explicit instruction of commas, colons and semicolons, *F*(1, 86) = 26.99, *p* < .001, partial eta squared = .24, and apostrophes, *F*(1, 86) = 17.67, *p* = .001, partial eta squared = .17. There were no significant differences by key stage for the other two punctuation items (all *p*s > .05). A mean score for the four punctuation items was computed, reflecting the frequency with which teachers reported focussing on punctuation. All the teachers reported focussing on punctuation and, on average, this was occurring virtually weekly, but there was large variation across the respondents (M = 2.89, SD = 1.04). There were no responses to the open-ended option in this section.

**Table 2:** Mean (SDs) of teachers’ reported frequency for teaching punctuation (0 = not taught to 5 taught daily)

Explicit instruction	Reception and KS1 (*n* = 37)	Key stage 2 (*n* = 51)	Total (N = 88)	Percentage reporting not taught
Punctuation at the end of sentences	4.43 (1.07)	4.47 (0.88)	4.45 (0.96)	0
Commas, semi-colons and colons*	1.76 (1.21)	3.22 (1.36)	2.60 (1.48)	1.1
Apostrophes to mark possession and omission*	1.62 (1.04)	2.80 (1.47)	2.31 (1.43)	1.1
Use of speech marks	2.03 (1.40)	2.31 (1.49)	2.19 (1.45)	10.2

* Significant differences in teachers’ reported frequency of teaching for the two groups of students, *p* < 0.01

#### Text generation

*Word*-*level work* Four items examined teachers’ focus on vocabulary in relation to the production of written text, and Cronbach’s alpha was .74 for these items. Table 3 provides means (SDs) by key stage for reported word-level foci. As the table shows, focussing on word-level work was a regular occurrence, with ‘the use of a wide range of vocabulary in an inventive way’ reported to occur several times a week while ‘using contrasts to highlight differences/similarities between words’ occurred virtually weekly. An initial MANOVA examined year group differences across the word-level items. There was a statistically significant difference in teachers’ reported focus by year groups taught, *F*(4, 83) = 3.13, *p* = .02; Wilk’s Λ = 0.87, partial eta squared = .13. Subsequent univariate testing indicated that teachers in KS2 reported a greater focus on the teaching of word classes and the grammatical function, *F*(1, 86) = 8.81, *p* = .004, partial eta squared = .09. There were no significant differences by key stage for the other three items (all *p*s > .05). A mean score for the four word-level items was computed. On average, word-level work was occurring several times a week but there was large variation across the respondents (M = 3.56, SD = 1.9), and one teacher reported doing no word-level work at all with students. There were no responses to the open-ended option in this section.

**Table 3:** Mean (SDs) of teachers’ reported frequency for teaching at word, sentence and text level (0 = not taught to 5 taught daily)

	Reception and KS1 (*n* = 37)	Key stage 2 (*n* = 51)	Total (N = 88)	Percentage reporting not taught
*Word level*				
Wide range of vocabulary in inventive ways	3.84 (1.30)	4.31 (1.05)	4.11 (1.18)	3.4
Contrasts that highlight differences/similarities between words	2.46 (1.61)	3.18 (1.37)	2.88 (1.51)	10.2
Expand and extend their vocabulary in written tasks by linking to prior knowledge	3.62 (1.53)	4.00 (1.04)	3.84 (1.28)	4.5
Teach word classes and the grammatical function of words*	2.86 (1.72)	3.78 (1.19)	3.40 (1.50)	6.8
*Sentence level*				
Highlight features of different types of sentences*	2.41 (1.40)	3.35 (1.31)	2.95 (1.42)	5.7
Explicit instruction in complex sentence grammar*	2.62 (1.53)	3.55 (1.39)	3.16 (1.52)	5.7
Draw students attention to differences in meaning between specific grammatical structures*	2.41 (1.64)	3.35 (1.25)	2.95 (1.49)	9.1
*Text level*				
Analyse forms of texts*	2.59 (1.34)	3.31 (1.21)	3.01 (1.31)	8.0
Teacher reads own writing	3.65 (1.36)	3.63 (1.30)	3.64 (1.31)	3.4
Require students to vary the formality of written language	2.57 (1.09)	2.76 (1.29)	2.68 (1.21)	2.3
Teach students to make choices in relation to topics and ideas	3.16 (1.57)	3.18 (1.73)	3.17 (1.66)	14.8
Instruction in paragraph construction and the linking of ideas*	2.14 (1.44)	3.25 (1.28)	2.78 (1.45)	8.0

* Significant differences in teachers’ reported frequency of teaching for the two groups of students, *p* < 0.01

*Sentence*-*level work* There were three items that examined teachers’ focus on sentence-level work, and Cronbach’s alpha was .80 for these items. Table 3 provides means (SDs) by key stage for reported sentence-level work. Sentence-level work was reported to occur weekly. An initial MANOVA examined year group differences across the sentence level items. There was a statistically significant difference by year groups taught, *F*(3, 84) = 4.37, *p* = .007; Wilk’s Λ = 0.87, partial eta squared = .13. Subsequent univariate testing indicated that teachers in KS2 reported a greater focus on all aspects of sentence-level work: drawing attention to differences in meaning between specific grammatical structures, *F*(1, 86) = 9.49, *p* = .003, partial eta squared = .10, highlighting different types of sentences, *F*(1, 86) = 10.58, *p* = .002, partial eta squared = .11, and explicit instruction in complex sentence grammar, *F*(1, 86) = 8.75, *p* = .004, partial eta squared = .09. A mean score for the three sentence-level items was computed. On average, sentence-level work was occurring weekly but there was large variation across the respondents (M = 3.02, SD = 1.28), and two teachers reported doing none with their students. There were no responses to the open-ended option in this section.

*Text*-*level work* There were five items that focussed on text-level work, and Cronbach’s alpha was .70 for these items. Table 3 provides means (SDs) by key stage for reported text-level work. Across the items text-level work was reported to occur weekly. An initial MANOVA examined year group differences across the text-level items. There was a statistically significant difference by year groups taught, *F*(5, 82) = 4.41, *p* = .001; Wilk’s Λ = 0.79, partial eta squared = .21. Subsequent univariate testing indicated that teachers in KS2 reported a greater focus on analysis of text forms, *F*(1, 86) = 6.92, *p* = .01, partial eta squared = .07, and paragraph construction, *F*(1, 86) = 14.82, *p* < .001, partial eta squared = .15. There were no significant year group differences for the three remaining text-level items (all *p*s > .05). A mean score for the five text-level items was computed. On average, text-level work was occurring weekly but there was large variation across the respondents (M = 3.06, SD = .92), and one teacher reported doing none at all. There were two respondents who provided additional information. One respondent stated that all activities were done but it depended on the topic, while the second respondent stated ‘repeating story language and using actions to retell stories to help their communication and language’.

#### Executive functions

*Planning, reviewing and revising* There were seven items that focussed on planning, reviewing and revising, and Cronbach’s alpha was .79 for these items. Table 4 provides means (SDs) by key stage. As the table shows there was marked variability across the items, but in general some aspects of planning, reviewing and revising were reported monthly. An initial MANOVA examined year group differences across the items. There was a statistically significant difference by year groups taught, *F*(7, 80) = 4.80, *p* < .001; Wilk’s Λ = 0.70, partial eta squared = .30. Subsequent univariate testing indicated that there were significant differences for all items except prepare a neat and final copy. In all cases these activities were more likely to occur in KS2: plan, note and develop initial ideas on paper, *F*(1, 86) = 7.64, *p* = .007, partial eta squared = .08, draft on computer, *F*(1, 86) = 7.46, *p* = .008, partial eta squared = .08, develop ideas from the plan into structured written text, *F*(1, 86) = 7.44, *p* = .008, partial eta squared = .08, create a handwritten draft before a word-processed draft, *F*(1, 86) = 8.84, *p* = .004, partial eta squared = .09, revise, *F*(1, 86) = 33.33, *p* < .001, partial eta squared = .28, and proof read, *F*(1, 86) = 12.57, *p* < .001, partial eta squared = .13. Only one teacher reported not doing any of the planning, reviewing and revising activities. On average planning, reviewing and revising was occurring monthly (M = 2.13, SD = .88).

**Table 4:** Mean (SDs) of teachers’ reported frequency for teaching planning, reviewing and revising (0 = not taught to 5 taught daily)

	Reception and KS1 (*n* = 37)	Key stage 2 (*n* = 51)	Total (N = 88)	Percentage reporting not taught
Plan, note and develop initial ideas on paper*	2.49 (1.30)	3.16 (0.97)	2.88 (1.16)	3.4
Students complete a rough draft on computer before producing a handwritten version*	0.73 (0.84)	1.31 (1.09)	1.07 (1.03)	27.3
Draft—develop ideas from the plan into structured written text*	2.00 (1.39)	2.75 (1.16)	2.43 (1.31)	10.2
Encourage students to create a handwritten draft before a word processed draft*	1.05 (1.08)	1.75 (1.07)	1.45 (1.12)	13.6
Revise—change and improve the draft*	1.65 (1.34)	3.20 (1.17)	2.55 (1.45)	10.2
Proofread—check the draft for spelling and punctuation errors, omissions and repetitions*	2.27 (1.69)	3.43 (1.37)	2.94 (1.61)	10.2
Present—prepare a neat, correct and clear final copy	1.49 (1.35)	1.76 (1.18)	1.65 (1.25)	8.0

* Significant differences in teachers’ reported frequency of teaching for the two groups of students, *p* < 0.01

Six respondents provided further information in the open-ended section: two respondents stated children proof read written work, two mentioned the children reading back their own work, one mentioned using whiteboards to encourage children to write, and one listed pictorial planning, photo sequencing of an activity, story mountain and brainstorming.

**Fig. 2:**
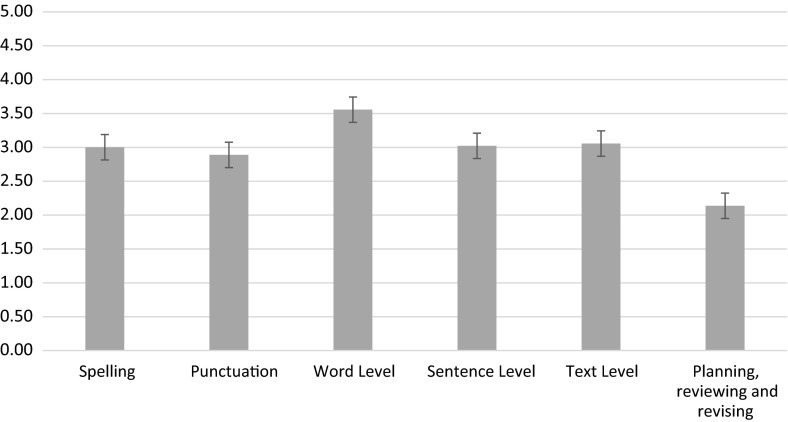
Mean (SE) of reported frequency of teaching across domains of writing (0 = not taught to 5 taught daily)

*Capturing the simple view of writing* A repeated measures ANOVA with key stage as the between measures variable and domains of teaching^3^ as the repeated measures variable examined the frequency with which the different domains of teaching were reported to be taught. Means and standard errors are reported in Fig. 2. There was a significant effect of domain, *F*(1, 86) = 71.24, *p* < .001, partial eta squared = .45, but no interaction by year group, *F*(1, 86) = 2.46, *p* = 12, partial eta squared = .03. Word-level work was reported significantly more than all other domains (all *p*s < .001), and planning, reviewing and revising was reported to occur significantly less than work in all other domains (all *p*s < .001).

### Reported teaching practices to support writing development

Teachers reported using, at least weekly, tools such as interactive white boards (M = 4.83, SD = 0.62), small white boards (M 4.63, SD = 0.70) and visual aids (M = 4.73, SD = 0.71) to support their teaching. There was, however, much greater variability in their pedagogical approaches to supporting children’s’ development of written texts.

Table 5 presents the teachers’ ratings for strategies used in the different key stages. Typically these activities occurred weekly, with modelling writing strategies being the most frequently reported strategy and using structured worksheets the least frequent. An initial MANOVA examined year group differences across the teaching practices reported. There was a statistically significant difference by year groups taught, *F*(1, 71) = 3.41, *p* < .001; Wilk’s Λ = 0.65, partial eta squared = .36. Subsequent univariate testing indicated that teachers in KS2 reported more frequent use of having students assess each other’s work, *F*(1, 71) = 18.75, *p* < .001, partial eta squared = .21. However, as with other practices there was significant variation in responses especially for the use of sentence combining.

**Table 5:** Mean (SD) of teachers’ reported strategies for supporting writing (0 = not used to 5 used daily)

	Reception and KS1 (*n* = 37)	Key stage 2 (*n* = 51)	Total (N = 88)	Percentage reporting does not occur
Peer assessment*	2.78 (1.36)	3.88 (1.01)	3.42 (1.28)	1.1
Structured worksheets	3.00 (1.18)	2.67 (1.54)	2.81 (1.40)	10.2
Sentence combining	2.92 (1.62)	3.43 (1.68)	3.22 (1.66)	13.6
Learn and rehearse texts	3.22 (1.34)	3.43 (1.24)	3.34 (1.28)	3.4
Discuss and evaluate own and/or others’ writing	3.41 (1.07)	3.67 (1.80)	3.56 (1.53)	11.4
Prior brainstorming to create visual map	3.51 (1.17)	3.86 (1.04)	3.72 (1.10)	1.1
Sentence or story starter	3.43 (1.30)	3.02 (1.57)	3.19 (1.47)	10.2
Constructing texts with students	4.11 (0.99)	4.02 (1.03)	4.06 (1.01)	1.1
Model a piece of writing, explaining vocabulary choices	4.22 (1.11)	4.24 (0.93)	4.23 (1.00)	2.3
Model writing strategies with small groups of children	4.43 (0.73)	4.29 (0.99)	4.35 (0.88)	1.1

* Significant differences in teachers’ reported frequency of teaching for the two groups of students, *p* < 0.01

Teachers were asked to report on any specialised writing packages that they used in their classrooms. There were 36 responses reflecting 14 different packages. Only one respondent mentioned a handwriting programme. The majority (28 %) were phonics programmes that included spelling/written activities, with two being mentioned [Jolly phonics (Jolly Learning) and Letters and sounds (Department for Education and Skills)]. The two programmes mentioned by four or more respondents focussed on text production [Read Write Inc (Oxford University Press) and Talk for Writing (www.Talk4writing.co.uk)]. Only one or two respondents mentioned the remaining 10 programmes.

## Discussion

There are continued concerns about students’ ability to produce written text in the UK, USA and Europe (Department for Education, 2013; Persky, Daane, & Jin, 2003; Torrance et al., 2012). Using the simple view of writing as a framework, supplemented by current English curricular guidance for writing, we examined instructional practices for writing using an online survey. Teachers in the sample were working with students who were both more disadvantaged than the national average and who were performing significantly below the national average in terms of their writing performance. Despite this, and in contrast to previous studies (e.g. Graham et al., 2014), the respondents in this study felt prepared to teach writing^4^ and they enjoyed teaching writing, but many reported that resources were not available. The clear and explicit guidelines for the teaching of writing in England arguably provides teachers with a framework which they feel prepares them to teach writing. However, the struggles that their pupils experience and the reported lack of resources raise concerns about the effective teaching of writing.

Teachers (41 % of the sample, *n* = 36) also provided information about the writing packages they were using. A broad range of packages was identified, but apart from phonic programmes which included spelling, there was little consistency in programmes that respondents reported they used. Teachers also felt they were effective in identifying approaches to support students’ writing. Despite these positive views about preparation and enjoyment of teaching writing, nearly half reported that supporting struggling writing was problematic for them; although those who had attended specialist training were significantly less likely to report that this was an area of concern for them. Given that a significant proportion of the children in these teachers’ classes were not reaching national standards for writing, this places significant demands on teachers’ abilities to differentiate the curriculum and provide instruction of sufficient intensity to meet their students’ needs. To address gaps in our current knowledge about teaching practices for writing in English schools, we asked teachers to report on what they taught and how often these activities occurred.

Handwriting practice was reported to occur at least weekly, whereas practice in typing and drafting texts on computers was a much rarer occurrence. This result is of concern given suggestions that technology should be made more integral to the teaching of writing (National Commission on Writing, 2003), and evidence that computers can enhance the quality of children’s writing (Graham & Perin, 2007a). The data suggest that children are receiving insufficient writing practice with new technology to support the development of their written texts. Given the significant proportion of children in these classes who were struggling with writing, there is clearly scope for examining the potential to use computers to enhance children’s writing in English schools (Rogers & Graham, 2008).

We created summary variables that captured elements of the simple and not so simple view of writing (Berninger & Amtmann, 2003; Berninger et al., 2002; Berninger & Winn, 2006). Overall teachers reported more work at word level, occurring several times a week, than at transcription, text or sentence level, which were reported to occur weekly. Planning, reviewing and revising was reported to occur least often, only monthly, which is consistent with the results of Graham et al. (2003) where less frequent attention was given to planning, reviewing and revising. By contrast the emphasis on word-level work is more prominent than in other studies (see for example Cutler & Graham, 2008) and suggests that teachers in these classrooms in England were using vocabulary activities to support writing. This is important given the increasing evidence showing how oral language can underpin written language (McCutchen, Stull, Herrera, Lotas, & Evans, 2014; Mehta, Foorman, Branum-Martin, & Taylor, 2005; Wagner et al., 2011); one of the key drivers to support text generation appears to be vocabulary (Babayiğit, 2015; Dockrell & Connelly, 2015). Our respondents placed less focus on planning, reviewing and revising compared to other areas, and this may indicate a gap in current teaching practices. Planning and drafting introduces children to the process of writing and needs explicit support from the teacher (Graham & Perin, 2007b). For all these summary aspects of the simple view of writing no differences in emphasis across the key stages were evident.

When specific aspects of each domain were examined both greater variation in teaching frequency and differences across key stages were evident. Teachers of older students focussed more on complex aspects of spelling such as word roots, punctuation such as commas, colons and semi colons, and the teaching of word classes and grammatical function of words. There was also a greater focus for all sentence level items and for paragraph construction with the older students. The frequency of what was taught also varied within and between aspects of the writing process. Sounding out phonemes, teaching about punctuation at the ends of sentences and using a wide range of vocabulary in inventive ways occurred most frequently.

In terms of intensity of instruction, the only activity that was reported to occur daily was sounding out phonemes for the younger cohort; this is not surprising given that the English national phonics test occurs at this point. Both sentence and text-level work occurred weekly for the older cohort but typically only monthly for the younger cohort. By contrast, activities related to planning, reviewing and revising tended to happen at most monthly, apart from proof reading, which happened at least weekly for the older cohort. These reports differ from other studies where, for example, spelling, grammar, capitalisation and punctuation were reported to occur daily in Grades 1–3 in the US (Cutler & Graham, 2008). There was also significant variation between respondents in their responses to specific items. As an example, consider teachers’ reports of providing explicit instruction in the use of appropriate terminology for spelling where some teachers reported this to be occurring several times a week whereas others only reported this occurring several times a year. This raises important questions about what guides teachers to make decisions about the frequency of these activities. Does it reflect their training, their students, school guidelines or a combination of these factors? Cutler and Graham (2008) highlight the tension between what should be taught and how frequently it should be taught. They note, “It may not be enough to introduce teachers to new writing practices and encourage them to apply them. Efforts to inform writing instruction are likely to fall short if little attention is devoted to how frequently practices are implemented. This needs to be the focus of both preservice as well as in service professional development” (2008: p. 916).

Teachers also reported practices that they used to support their teaching of writing, and as with content of writing instruction there was significant variability in how often respondents reported to be engaged in these activities. The most common activities involved teachers working with students either to construct texts or to model texts or to engage in ‘brainstorming’. These activities were reported to occur several times a week and virtually no teacher reported not using them. Both prewriting activities and the use of models have been shown to be effective in enhancing children’s writing, but there are also more explicit activities such as sentence combining and strategy instruction that produce larger effect sizes (Graham & Perin, 2007b) and were less evident in the reports of the current cohort of teachers: 13 % of the teachers reported never using sentence combining. It is of concern that only one of the packages that the teachers reported using had been subject to a systematic evaluation (Jolly Phonics; Stuart, 1999). This may be less important for phonics where there is now a consensus that teaching systematic synthetic phonics in a highly structured and systematic way is effective for teaching reading (National Institute of Child Health and Human Development, 2000; Rose, 2006; Wyse & Goswami, 2008), but more problematic for generic programs which aim to support text production and lack an evidence base.

How often should teachers engage in these writing activities, what level of intensity is required to gain mastery? There has been a general plea that students should write more (National Commission of Writing, 2003), that this writing should include expository as well as narrative texts, and that teaching should be balanced between teaching basic skills, teaching writing strategies and processes and writing texts (Cutler & Graham, 2008). However, the amount of time that children should be engaged in these activities is uncertain.

There is evidence from other areas where children struggle to learn that both intensification of instruction and distributed learning is critical. Spaced versus massed learning consistently shows benefits whereas extended intervals between learning sessions can attenuate performance (Cepeda, Pashler, Vul, Wixted, & Rohrer, 2006). Spacing effects have also been demonstrated for vocabulary learning in primary school children (Goossens, Camp, Verkoeijen, Tabbers, & Zwaan, 2012). Translating these principles into the teaching of writing would suggest that teachers should be engaging in these practices regularly with short intervals between teaching sessions. While the current National Curriculum for English (Department for Education, 2013) advises frequent practice in letter formation for 7 year olds and opportunities to write for a range of audiences for 8 and 9 year olds there is no indication of what frequently means not how these opportunities should be provided. The question of how regularly and for how long remains an empirical question (but see Hier & Eckert, 2014, for an example of weekly feedback for supporting writing fluency).

We used the simple view of writing to frame the questions we asked teachers. Operationalising the framework in this way has highlighted a number of issues which require clarification and development. Comparing teaching practices to a model can highlight missing emphasis in teaching but also, potentially, identify skills which are missing from the model. It was evident when we mapped the model on to teaching practices there were a number of inconsistencies. Specifically the model does not explicitly include punctuation, a core area of the curriculum and a skill teachers in England target regularly. We followed Hayes and Olinghouse (2015) in including it in transcription. However, punctuation also supports coherence and the presentation of complex ideas. Similarly while text generation is related to the communicative aspects of writing there is a lack of specificity of which aspects should be included here. The model also appears to compartmentalise aspects of writing without addressing how those different aspects interact with one another at different points in the development of children’s writing skills. For example, morphemes provide information about both a word’s meaning and its spelling. So although the model captures many of the core aspects of the writing process, the level may be too general to inform instruction. Indeed, Hayes and Olinghouse (2015) highlight that the teaching of writing should be considered in the light of various different models of writing rather than just a single model, because different models can provide different perspectives on teaching.

## Limitations

We had complete data from 88 teachers representing 10 schools as we excluded respondents who had omitted to respond to individual items. This was an e-survey and data were anonymous so it was not possible to calculate what percentage of teachers from the schools actually completed the survey, nor was it possible to make between-school comparisons. Teachers may have chosen to respond because of a particular interest in writing or because they felt they were competent teachers of writing, although we have no reason from the data related to teacher demographics to confirm this.

Students were not representative of national proportions for free school meals (our sample contained a higher proportion than the national average), special educational needs (a higher proportion that the national average) and English as an additional language (a lower proportion than the national average). These different demographic features may influence both what teachers taught and how they taught. For example, teachers of more competent writers might be predicted to focus on sentence or text level work rather than word level work (see for example Myhill, Jones, Lines, & Watson, 2012).

We reasoned that respondents understood the linguistic terminology of the survey questions. Terms such as “orthographic patterns”, “word families, roots and origins” and “suffixes and prefixes” were included in the survey because they are used in the National Curriculum, but that of course is not a guarantee that teachers actually understand them.

In collecting teachers’ reports of their practices we have relied, as others have done (Cutler & Graham, 2008; Gilbert & Graham, 2010), on teachers accurately reporting what they do in the classroom. Although there is indicative evidence that teachers’ responses reflect their practice (see Cutler & Graham, 2008; Gilbert & Graham, 2010) future studies should consider triangulating reports, classroom observations and pupils’ writing products. These studies should also consider explicitly the relationship between input, learning and what subsequently happens (Parr & Timperley, 2010).

Together the limitations of the current study indicate that further work is needed with a nationally representative sample of teachers across schools and key stages. These studies should include information about the amount of time teachers spend on these teaching activities, the nature of the writing tasks children are engaged in that is expository or narrative texts (Dockrell, Connelly, Walter, & Critten, 2014; Olinghouse & Wilson, 2013) and the extent to which children are engaged in extended writing across other areas of the curriculum.

## Summary

The current study reports the first survey of primary school teachers in England teaching of writing. In contrast to previous studies, the teachers reported being well trained to teach writing. Teachers covered the majority of the components of the writing process but focussed more on word-level work, while maintaining regular attention to spelling and end of sentence punctuation. The only aspect of writing that was reported to occur daily was phonic activities related to spelling for the younger cohort. These results contrast with other studies where spelling, grammar, capitalisation and punctuation were reported to be taught daily (Cutler & Graham, 2008). The focus on phonics and word-level work is likely to reflect government guidelines on the teaching of reading. It might be predicted with the new emphasis on the teaching of grammar teachers’ priorities may change to meet national targets. In addition to supporting teachers in using developmental models and effective teaching practices, there is a pressing need for researchers to establish the amount and regularity of teaching required to support the different components of the writing process, and to explicitly include in their models all the aspects of writing that teachers focus on in the classroom.

## Footnotes

1 Ofsted is the Office for Standards in Education, Children's Services and Skills in England. Ofsted inspect and regulate services that care for children and young people, and services providing education and skills for learners of all ages.

2 Receipt of free school meals was the metric of disadvantage used by the UK government at that time. This has now been replaced by the pupil premium https://www.gov.uk/pupil-premium-information-for-schools-and-alternative-provision-settings.

3 As the handwriting and typing domain only contained two items with statistically significant differences in frequency this domain was not included in the analysis.

4 59 % of our respondents reported their training to teach writing as either “very good” or outstanding”, which is similar to the figure of 55 % in Darling-Hammond et al. (2002) for US teachers reporting on their preparation for teaching different subject matter, concepts, knowledge and skills more generally.
